# A pragmatic randomised controlled trial of hydrotherapy and land exercises on overall well being and quality of life in rheumatoid arthritis

**DOI:** 10.1186/1471-2474-8-23

**Published:** 2007-03-01

**Authors:** Lis Eversden, Fiona Maggs, Peter Nightingale, Paresh Jobanputra

**Affiliations:** 1 Department of Physiotherapy, Selly Oak Hospital, University Hospital Birmingham NHS Foundation Trust, Raddlebarn Road, Birmingham, B29 6JD, UK; 2 Department of Rheumatology, Selly Oak Hospital, University Hospital Birmingham NHS Foundation Trust, Raddlebarn Road, Birmingham, B29 6JD, UK; 3 Wellcome Trust Clinical Research Facility, Queen Elizabeth Hospital, University Hospital Birmingham NHS Foundation Trust, Birmingham, B15 2TH, UK

## Abstract

**Background:**

Hydrotherapy is highly valued by people with rheumatoid arthritis yet few studies have compared the benefits of exercises in heated water against exercises on land. In particular, data on quality of life is rarely reported. This is especially important because patients treated with hydrotherapy often report an enhanced sense of well-being. We report a randomised controlled trial in which we compared the effects of hydrotherapy with exercises on land on overall response to treatment, physical function and quality of life in patients with rheumatoid arthritis.

**Methods:**

One hundred and fifteen patients with RA were randomised to receive a weekly 30-minute session of hydrotherapy or similar exercises on land for 6 weeks. Our primary outcome was a self-rated global impression of change – a measure of treatment effect on a 7-point scale ranging from 1(very much worse) to 7 (very much better) assessed immediately on completion of treatment. Secondary outcomes including EuroQol health related quality of life, EuroQol health status valuation, HAQ, 10 metre walk time and pain scores were collected at baseline, after treatment and 3 months later. Binary outcomes were analysed by Fisher's exact test and continuous variables by Wilcoxon or Mann-Whitney tests.

**Results:**

Baseline characteristics of the two groups were comparable. Significantly more patients treated with hydrotherapy (40/46, 87%) were much better or very much better than the patients treated with land exercise (19/40, 47.5%), p < 0.001 Fisher's exact test. Eleven patients allocated land exercise failed to complete treatment compared with 4 patients allocated hydrotherapy (p = 0.09). Sensitivity analyses confirmed an advantage for hydrotherapy if we assumed non-completers would all not have responded (response rates 70% versus 38%; p < 0.001) or if we assumed that non-completers would have had the same response as completers (response rates 82% versus 55% p = 0.002). Ten metre walk time improved after treatment in both cases (median pre-treatment time for both groups combined 10.9 seconds, post-treatment 9.1 s, and 3 months later 9.6 s). There was however no difference between treatment groups. Similarly there were no significant differences between groups in terms of changes to HAQ, EQ-5D utility score, EQ VAS and pain VAS.

**Conclusion:**

Patients with RA treated with hydrotherapy are more likely to report feeling much better or very much better than those treated with land exercises immediately on completion of the treatment programme. This perceived benefit was not reflected by differences between groups in 10-metre walk times, functional scores, quality of life measures and pain scores.

## Background

Hydrotherapy, defined as supervised exercise in warm water, is valued highly by people with rheumatoid arthritis and yet provision of hydrotherapy in Britain continues to decline [1]. All forms of exercise improve function and well-being in RA and concerns about disease exacerbation, even with intensive exercises, have not been borne out [2]. Indeed exercise is a key, and often ignored, risk factor for cardiovascular disease: increased physical activity in people with arthritis promises an important non-pharmacologic means of reducing cardiovascular disease [3].

Immersion in warm water reduces load on painful joints, promotes muscle relaxation and, with some fun, allows exercise against water resistance. A small number of previous randomised trials have examined the benefits of balneotherapy (bathing in warm water). All of these studies had methodological flaws and few compared the effect of exercises in warm water with exercises on land in RA [4,5]. Only one, a trial of hydrotherapy versus usual daily activities which included a total of forty six patients, assessed changes in quality of life: Bilberg and colleagues [6] found no difference in the physical component of the Short-Form 36 between hydrotherapy treated subjects and controls.

In this randomised controlled trial we set out to compare individualised exercises whilst immersed in a heated pool to similar exercises on land for their effect on overall improvement in health, physical function and quality of life in people with rheumatoid arthritis.

## Methods

### Participants

Men and women aged 18 years or older with RA (meeting American College of Rheumatology criteria), in functional classes I, II or III [7] and attending rheumatology clinics at Selly Oak Hospital in Birmingham were invited to participate either on referral for physiotherapy or by invitation in clinics or by mail. For inclusion, participants gave informed consent in writing and were required to understand and follow simple instructions in English. Patients needed to be on stable doses of disease modifying anti-rheumatic drugs (DMARDs) for 6 weeks and NSAIDs for 2 weeks before entry. Injections with corticosteroids in the 4 weeks before study entry were not permitted but drug changes and injections were permitted during the study to reflect the pragmatic nature of our study. Data on drug therapy was collected at baseline and when final assessments were made.

Patients who had had surgery in the 3 months before study entry or those who had surgery planned were excluded. Other exclusions were patients who had received physiotherapy or hydrotherapy in the 6 months preceding entry: this was done to avoid possible carry-over effects of previous therapy. Patients with known chlorine sensitivity, an infected open wound, poorly controlled epilepsy, hypertension, diabetes, incontinence of faeces, and a fear of water precluding hydrotherapy were excluded. Also excluded were: pregnant women; patients with co-morbid conditions which, in the opinion of the assessing physiotherapist, prevented safe use of hydrotherapy; known carriers of methicillin resistant staphylococcus aureus in the upper respiratory tract; and those who weighed more than 102 kg. The latter because of the safety procedures established for our pool.

A single senior physiotherapist assessed study eligibility in all cases. The Research and Development and Ethics committees of University Hospital Birmingham NHS Foundation Trust gave ethical approval for this study.

### Randomisation

Patients were randomised to hydrotherapy or land exercises using sealed opaque envelopes indicating treatment allocation. Randomisation envelopes were prepared at study inception and random number sequence was obtained by flipping a virtual coin [8]. A research assistant not involved in the conduct of the study randomised patients, allocated treatment and collected key data.

### Interventions

Participants, in groups, received either a weekly 30-minute session of hydrotherapy (at 35°C) or land based exercises for 6 weeks. Participants were asked to attend weekly but, allowing for sickness and leave, were allowed to default for up to three sessions as long as 6 sessions in all were completed. Those defaulting more than 3 sessions were considered to be treatment failures but were followed up, if possible, to obtain study data. Written instructions on home exercises were provided at the outset to all patients. Patients were not required to do exercises between treatment sessions but could do so if they chose. The physiotherapist who had assessed eligibility supervised treatment in both groups, with support from two other senior physiotherapists. A rolling treatment programme was operated for convenience, therefore group sizes varied between 1 to 4 for hydrotherapy and 1 to 6 for land exercises. The size of our hydrotherapy pool only permitted treatment of 4 patients at any one time. However group sizes in both treatment arms were comparable.

The exercise content in each group was similar and exercises were tailored to each individual's ability. Participants warmed up, by mobilising and stretching. The core exercises, repeated each week, focussed on joint mobility, muscle strength and functional activities. The degree of difficulty was reviewed weekly to ensure each participant made progress at their individual pace. A cool down phase concluded each session. Functional limitations of participants were considered at all times.

### Outcome measures

A research assistant blind to treatment allocation assessed key outcome measures. The primary outcome was self-rated overall effect of treatment, measured once; on the day treatment was completed. This validated measure has previously been used in clinical trials of exercise in fibromyalgia and chronic fatigue syndrome [9-11]. Effect of treatment is measured as change on a 7-point scale ranging from 1 (very much worse) to 7 (very much better). Patients were asked: 'please indicate how you feel after your treatment'. The outcome was dichotomised so that participants scoring 6 (much better) or 7 were regarded as responders and others as non-responders [10].

Secondary outcomes were collected at baseline, on the day of the last treatment session and 3 months post treatment. These included: pain, assessed on a 10 cm visual analogue scale (VAS), where 0 cm represented no pain; physical function assessed with the health assessment questionnaire (HAQ); ten metre walk speed, an outcome widely used to assess lower limb function in neurology [12] and piloted previously in our unit [13]; and a EuroQol-5D (EQ-5D) valuation questionnaire comprising a self report of health related quality of life (EQ-VAS) and a health status valuation (EQ-5D index or utility score) [14,15].

### Statistical considerations and analyses

Sixty patients were needed in each treatment group to detect a 28% difference between the two groups in the primary outcome measure with a two-sided significance level of 0.05, and a power of 80%, allowing for a 10% drop-out rate. This estimate was based on a pilot study showing that 73% of patients receiving hydrotherapy experienced an increased sense of well-being [13]. We assumed that around 45% of patients with RA undertaking land exercises would feel 'much better' or 'very much better' based on trials of exercise in other conditions [11]. Binary outcomes were analysed by Fisher's exact test and continuous variables by Wilcoxon signed rank tests for within group comparisons or by Mann-Whitney tests for between group comparisons. Data analyses were done according to the principles of intention to treat.

## Results

### Patient Characteristics and Disposition

**Figure 1:**
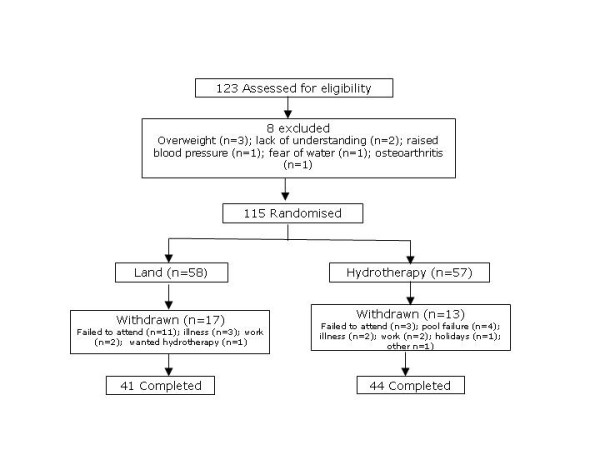
Study outline.

Our goal was to recruit 60 patients into each of the two treatment groups. However, recruitment was terminated early because of a decision by University Hospital Birmingham NHS Foundation Trust to close their hydrotherapy facility although this closure was deferred for several months to permit continuation of our study [1]. Three hundred and seventy patients (107 males and 263 females) were invited to participate in the study by mail. Others, an unknown number, were invited during routine clinic contacts. At termination, 123 patients had been assessed for eligibility, 8 were excluded and 115 randomised. Reasons for exclusion are shown in Figure 1. Fifty seven patients were allocated hydrotherapy. Data on the primary outcome was available for 46 (81%) hydrotherapy treated patients: two patients did not attend all follow-up visits and this data was obtained by mail. Fifty eight patients were allocated land exercises. Data on the primary outcome was available for 40 (69%) patients: one patient did not attend all follow-up visits and this data was obtained by mail.

Baseline characteristics of patients in the two groups were comparable (Tables 1 &2). Eleven patients allocated to land exercise failed to attend for the entire treatment programme compared with 4 patients allocated hydrotherapy (p = 0.09). Notably, one patient withdrew immediately from the study because he was allocated land exercises.

**Table 1:** Characteristics of patients at baseline.

Characteristic	Hydrotherapy N = 57	Land Exercise N = 58
Age (mean ± SD, years)	55.2 ± 13.3	56.1 ± 11.9
Female Sex	39 (68%)	42 (72%)
Disease Duration† (years)	10 (4–18)	8 (3–20)
On DMARD*	47 (82%)	50 (86%)
On oral corticosteroids	11 (19%)	10 (17%)
On NSAIDs	28 (49%)	32 (55%)

*DMARD includes TNF inhibitors but not corticosteroids.

† Values are medians with lower and upper quartiles in parentheses

**Table 2:** Secondary Outcomes†

	**Hydrotherapy**			**Land Exercises**		
**Outcome Measure**	Baseline N = 57	Immediately Post Treatment N = 44	3 mo. post treatment N = 43	Baseline N = 58	Immediately Post Treatment N = 40	3 mo. Post treatment N = 42

EQ-5D Utility	0.69 (0.52–0.80)	0.69 (0.59–0.78) p = 0.61	0.62 (0.52–0.76) p = 0.044	0.69 (0.59–0.76)	0.68 (0.59–0.79) p = 0.57	0.66 (0.52–0.76) p = 0.044
EQ-5D VAS (0–100)	70 (50–85)	73 (55–85)** p = 0.57	70 (44–80) p = 0.08	74 (60–86)*	77 (60–90) p = 0.42	75 (51–88)*** p = 0.63
HAQ (0–3)	1.38 (0.69–2.00)	1.50 (1.06–1.84) p = 0.09	1.63 (1.13–1.88) p = 0.23	1.50 (0.88–2.00)*	1.44 (0.78–1.84) p = 0.20	1.38 (0.84–1.91) p = 0.77
Pain (100 mm VAS, 0 = no pain)	24 (10–50)	25.5 (11–41) p = 0.40	35 (14–62) p = 0.026	26.5 (14–51)	27.5 (15–58) p = 0.22	42.5 (16–59) p = 0.005
10 m walk time (seconds)	10.9 (7.9–12.8)	9.1 (7.2–10.7) p < 0.001	10.0 (8.0–12.0)** p = 0.011	10.2 (8.8–13.2)	8.8 (6.9–11.3) p < 0.001	9.0 (7.7–11.3) p < 0.001

† Values are medians with lower and upper quartiles in parentheses and p values for comparison with baseline

* n = 57 ** n = 42 *** n = 41

### Primary outcome

Significantly more patients treated with hydrotherapy (40/46, 87%) felt much better or very much better than the patients treated with land exercise (19/40, 47.5%), p < 0.001 Fisher's exact test. Because a greater number of patients allocated land exercises failed to attend we tested the robustness of our data by doing some sensitivity analyses. First, we assumed that all non-completers would have been non-responders: this gave a response rate for hydrotherapy of 40/57 (70%) and for land 19/58 (33%), p < 0.001 in favour of hydrotherapy. Second, we assumed that non-completers would have had the same response rate as completers and that this proportion would have been the same for both treatment groups (null hypothesis). Thus 47/57 (82%) of hydrotherapy patients and 32/58 (55%) of land patients would have responded, p = 0.002. Finally, assuming that all non-completers in hydrotherapy would have been non-responders (40/57, 70%) and that all non-completers in the land group would have been responders (37/58, 64%), a rather implausible assumption, showed that there was no significant difference between the groups, p = 0.59.

### Secondary outcomes

Ten-metre walk time improved after treatment and gains were maintained 3 months after treatment in both cases (median pre-treatment time for both groups combined 10.9 seconds(s), post treatment 9.1 s and at 3 months post treatment 9.6 s). There was no difference between treatment groups (p = 0.551). Similarly, there were no significant differences between groups in terms of changes to HAQ, EQ-5D utility score, EQ VAS and pain VAS (Table 2). Three months after treatment pain scores were significantly increased compared with baseline values in both treatment groups (Table 2). Also, 3 months post treatment worsening of pain and of the self care domains of EQ-5D was also seen (p = 0.029 and p = 0.02 respectively; McNemar's test). In the domain of pain/discomfort 19/85 (22.4%) worsened by at least one category, 7 (8.2%) improved and 59 (69.4%) stayed the same. For self care 23/85 (27.1%) worsened, 9 (10.6%) improved and 53 (62.4%) stayed the same.

Medication changes occurred commonly during the trial. For example, at the final assessment, 3 months after completion of treatment, 10 (17.5%) patients treated with hydrotherapy had changed or increased the dose of their DMARD compared with 9 (15.5%) land patients; 4 (7%) hydrotherapy patients decreased or ceased their DMARD compared with 2 (3%) land; and 4 (7%) in both groups had had steroid injections.

## Discussion

We have shown that patients with RA undertaking exercises in a heated pool are significantly more likely to feel much better or very much better than patients doing similar exercises on land. We demonstrated this using a transitional outcome measure assessed after 6 weeks of exercises [10,11]. Patient reports of benefit were not reflected by health status measures of pain, HAQ scores, EQ-5D utility scores and self rated global health (EQ VAS). This is surprising because previous studies, done to validate EQ-5D utility and VAS scales, have shown these measures to be highly responsive to self reported improvement [16]. However measures such as HAQ may be less responsive, particularly in studies of exercise in RA [17]. The absence of benefit in health utility measures or in self rated global health scores indicates that hydrotherapy would not be judged cost effective because of the additional resources needed for hydrotherapy. A formal economic study of hydrotherapy for juvenile idiopathic arthritis, based in part at our institution, also concluded that hydrotherapy would not be cost-effective [18]. Indeed during the conduct of our trial our institution decided to close the hydrotherapy facility.

Our data underscores concerns about the relevance of health status measures such as EQ-5D to the personal perspectives of patients [19,20]. We assessed self reported change in health on the day treatment was completed but our primary outcome was not evaluated 3 months post treatment. This was an important limitation of our study. We believed, however, that it was important to capture the direct impact of exercises since our pragmatic study design did not restrict use of other interventions, including use of corticosteroids during the study. Later assessment of our primary outcome may have been confounded by recall bias and other interventions in the weeks after completion of exercises. Many patients changed their drug therapies or received steroid injections especially in the 3 months post treatment, although the proportions of patients changing therapy were similar in the two groups. A pragmatic design is important for aiding policy decisions about therapeutic resources [21]. We felt this design was important to assess the value of hydrotherapy in a realistic setting since treatments in chronic arthritis are commonly used as one component of a complex set of interventions.

Care was taken to ensure that the same therapist treated both groups of patients as far as was practical and indeed a majority of treatments in both arms were done by one person. Use of different therapists could have led to important differences in approach to patients' problems or in outcomes because of differences in the abilities of physiotherapists to motivate and heal patients. However, we acknowledge that a conscious or subconscious preference for one or other treatment by the treating therapist may have influenced outcomes. In order to improve internal validity (reliability of the result) outcomes were patient-centred and an independent assessor collected outcomes. We also ensured that group sizes were similar so that therapeutic benefits from being part of a group were similar. It is known that, in addition to the physiological and cardiovascular benefits of exercise, exercise classes also provide opportunities for socialisation and mutual support, and that both are important determinants of continued exercise [22].

Failure to adhere to exercises is a common human failing mirrored in people with arthritis [23]. Some suggest that compliance with exercises in inflammatory arthritis is better in those who report greater benefits of exercise[24] and others that even short term interventions can have long term beneficial effects [25]. Certainly both groups showed a sustained improvement in walk times but pain scores were increased 3 months after treatment in both groups. Whether this reflected disease progression, greater pain scores because of increased physical activity, or other factors, is unknown. Other studies of hydrotherapy and exercise in arthritis either report improved pain [6] or no effect of exercise on pain [2,18] or disease activity [17].

## Conclusion

Our study clearly shows that RA patients who attend hospital clinics are more likely to report feeling much better or very much better if they are treated with hydrotherapy than if they are treated with exercises on land. This benefit was reported immediately after completing treatment. Whether this gain is sufficient to justify provision of hydrotherapy facilities in a hospital setting is debatable. For ambulatory patients with arthritis provision of aquatic exercise through community initiatives may be more effective in terms of public health [26]. We cannot say whether hydrotherapy offers advantages over other forms of intensive rehabilitation to people with severe disability; this remains to be explored.

## Competing interests

The author(s) declare that they have no competing interests.

## Authors' contributions

PJ and LE conceived, planned and secured funding for the study. LE carried out and supervised treatments and ensured that measurements of key outcomes were consistent. PN did the data analyses and sample size calculations. FM communicated with patients including treatment allocation, collected key data and maintained the database. PJ co-ordinated the study and drafted this manuscript. All authors read and approved the final manuscript.

## Pre-publication history

The pre-publication history for this paper can be accessed here:


